# Evidence for water deficit-induced mass increases of raffinose family oligosaccharides (RFOs) in the leaves of three *Craterostigma* resurrection plant species

**DOI:** 10.3389/fphys.2015.00206

**Published:** 2015-07-22

**Authors:** Aurélie Egert, Barbara Eicher, Felix Keller, Shaun Peters

**Affiliations:** ^1^Institute of Plant Biology, Molecular Plant Physiology, University of ZürichZürich, Switzerland; ^2^Department of Genetics, Institute for Plant Biotechnology, University of StellenboschStellenbosch, South Africa

**Keywords:** raffinose oligosaccharides, resurrection plants, desiccation tolerance, craterostigma species

## Abstract

The leaves of the resurrection plant *Craterostigma plantagineum* accumulate sucrose during dehydration, via a conversion from the unusual C8 ketose-sugar 2-octulose. However, raffinose family oligosaccharides (RFOs) have been shown to be major photosynthetic products in this plant. The tetrasaccharide stachyose is the major phloem-mobile carbohydrate and is used as a carbon store in roots. It has been suggested that this carbon store is remobilized during rehydration, presumably for cellular repair processes. We examined the effects of water deficit on the leaf water-soluble carbohydrate profiles of three *Craterostigma* species. Apart from the classical 2-octulose-to-sucrose interconversion, there was a strong water deficit-associated mass increase of RFOs up to the pentasaccharide verbascose. However, the activities of three dedicated RFO biosynthetic enzymes (raffinose, stachyose, and verbascose synthase) was not correlated with RFO accumulation, suggesting that biosynthetic enzyme activities measured in the early stages of water-deficit were sufficient to synthesize enough galactinol and lead to RFO accumulation in the leaves. Our findings are suggestive of RFOs providing additional carbohydrate-based stress protection to the leaves of these plants during the desiccated state.

## Introduction

The vegetative tissues (leaves and roots) of most angiosperm plants are unable to survive water deficit beyond a 60% loss of their relative water content (RWC). However, the so-called resurrection plants display the rare phenomenon of being truly desiccation tolerant, able to survive near complete loss of water (>90% RWC) by effectively remaining in metabolic stasis and resuming normal cellular metabolism within a short period after water has become available again (for reviews see Gaff, 1989; Farrant, 2000; Scott, 2000; Vicré et al., 2004). Only some 330 angiosperm species have been described to be “resurrection competent” (Proctor and Pence, 2002).

The dicotyledonous South African resurrection plant *Craterostigma plantagineum* (Hochst) has, historically, been the main model for studying desiccation tolerance in plants at both the molecular and physiological level (Bartels and Salamini, 2001; Bartels, 2005; Rodriguez et al., 2010). One of its most striking physiological features is the accumulation of the unusual C8 ketose-sugar 2-octulose (Oct) in the leaves at full turgor. This has been reported to account for up to 90% of the total water soluble carbohydrates (WSCs), corresponding to up to 400 mg g^−1^ of lyophilized leaf material (Bianchi et al., 1991).

Amongst the most notable changes occurring in the leaves of *C. plantagineum* undergoing water deficit stress is a remarkable, and reversible, interconversion between Oct and sucrose (Suc). It has been proposed that transketolases (key enzymes of the reductive and oxidative pentose phosphate pathways, responsible for the synthesis of sugar phosphate intermediates) contribute to the Suc-to-Oct interconversion during rehydration of *C. plantagineum* and at least two transketolase genes have been demonstrated to be transcriptionally upregulated during rehydration (Bernacchia et al., 1996). The accumulation of Suc appears to be a ubiquitous response to water deficit stress in the leaves of resurrection plants, but as is the case with *C. plantagineum*, different routes may be used for this accumulation.

Whilst Oct is the predominant WSC in un-stressed leaves, the predominant WSC in both stressed and un-stressed roots is the raffinose family oligosaccharide (RFO) tetrasaccharide, stachyose (Sta, >50% of total root WSC; Norwood et al., 2003). Further, both Oct and Sta have been reported to be major phloem-mobile WSCs in *C. plantagineum* (Norwood et al., 2000). However, Oct occurs only in trace amounts in the roots, suggesting that Sta is the predominant carbon store in roots. During water deficit stress, Sta in the roots is mobilized to Suc, but has been suggested to be translocated to other areas of the plant to support carbohydrate metabolism during dehydration of the tissue (Norwood et al., 2003).

There are no reports of water deficit-induced changes in RFOs in the leaves of *C. plantagineum*. This is surprising, given that RFOs are frequently suggested to contribute to desiccation tolerance and that *C. plantagineum* produces RFOs in un-stressed leaves as photosynthetic products (Norwood et al., 2000). The ability of RFOs to protect molecular structures has been extensively demonstrated *in vitro* (Crowe et al., 1992; Hoekstra et al., 2001). Within the context of resurrection plants, RFOs may be effective in either (i) directly, protecting cellular components during desiccation or (ii) indirectly providing protection by preventing the crystallization of water deficit-induced Suc in the desiccated tissue.

In this study, we identified a series of WSCs from leaves of desiccated (5% RWC). *C. plantagineum* which showed the same retention time, after HPLC separation, as an RFO series of standard oligosaccharides (Raf, Sta, and Ver). We confirmed their identity enzymatically as being *bona fide* RFOs. To further analyse the occurrence of these RFOs in the leaves, we subjected *C. plantagineum* plants to soil-drought and quantified the changes in WSCs and the corresponding RFO biosynthetic enzyme activities (Gol synthase, GolS, Raf synthase, RafS, and Sta synthase, StaS), over the drought period. As reported previously, Oct concentrations declined in the leaves concomitant to increases in the Suc concentrations. Additionally we observed increases in RFO concentrations, particularly the tetrasaccharide Sta. Further comparison of leaf WSC concentrations between the three *Craterostigma* species, *C. plantagineum, C. agnewi*, and *C. pumilum*, in the fully hydrated state and the dried state confirmed that this RFO increase during dehydration is common for the three *Craterostigma* species tested. Our findings suggest that this *de novo* water deficit-induced RFO biosynthesis may provide additional carbohydrate protection in desiccated leaves.

## Materials and methods

### Plant material and stress conditions

*Craterostigma* plants were maintained under glasshouse conditions (Bachmann and Keller, 1995; Peters et al., 2007) at the botanical gardens (University of Zürich, Switzerland). Prior (30 d) to any experimentation, plants were transferred to controlled environment conditions (12 h light, 30 μmol photons m^−2^ s^−1^, 22°C, 12 h dark, 60% RH). Water deficit was imposed on soil-grown plants as previously described (Peters et al., 2007). RWC was calculated using the formula of Barrs and Weatherley (1962):

$$\begin{array}{r} \text{RWC}=\left[\left({\text{W}}_{\text{i}}-{\text{W}}_{\text{d}}\right)∕{\text{W}}_{\text{d}}\right]∕{\left[\left({\text{W}}_{\text{t}}-{\text{W}}_{\text{d}}\right)∕{\text{W}}_{\text{d}}\right]}^{*}100 \end{array}$$

### Leaf crude enzyme extracts

Crude enzyme extracts were prepared by grinding freshly harvested leaf material (two discs, 6 mm diameter) in 500 μl of chilled extraction buffer as previously described (Peters et al., 2007). Aliquots (20 μl) of desalted extract were assayed for GolS activity in a final volume of 40 μl assay buffer containing final concentrations of 50 mM *myo*-inositol and 5 mM UDP-galactose, at 30°C for 30 min. RafS and StaS activities were assayed at 30°C for 60 min in the same volumes with 10 mM Gol and 100 mM Suc and Raf, respectively. The corresponding products, Gol, Raf, and Sta, respectively, were analyzed by HPLC-PAD as described below.

### Enzymatic hydrolysis of RFOs

Fractions representing a series of peaks eluting at the same retention time as RFO standard sugars (Raf, Sta, Ver) were collected after separation on a BC-100 column, prior to the addition of post-column NaOH. The fractions were then hydrolyzed with a fungal acidic α-galactosidase and re-analyzed as previously described (Peters et al., 2007).

### Soluble carbohydrate extraction

Ground, freeze-dried leaf material (25 mg) was used to extract WSCs as previously described (Peters et al., 2007). Aliquots (50 μl) were desalted and analyzed by HPLC-PAD as described below.

### Desalting of extracts

Desalting of WSC and enzyme assay samples to remove phenolic and charged compounds was conducted by centrifuge-rinsing of the samples through pre-rinsed 1 ml Mobicol spin columns (MoBiTec, Göttingen, Germany), fitted with a 10 μm frit as previously described (Peters et al., 2007; Peters and Keller, 2009).

### HPLC-PAD analysis and quantification of carbohydrates

WSCs were separated and quantified from plant extracts and enzyme assays by HPLC-PAD as previously described (Bachmann et al., 1994; Peters et al., 2007; Peters and Keller, 2009). Briefly, The BC-100 HPLC system comprised a Ca^2+^/Na^+^ moderated ion partitioning carbohydrate column (Benson BC-100 column, 7.8 × 300 mm; Benson Polymeric, Reno, Nevada, USA) operated at 90°C and isocratically eluted with 0.005% (w/v) Ca/Na_2_-EDTA at a flow rate of 0.6 ml min^−1^. To confirm the identities of certain carbohydrates, samples were also analyzed by anion exchange chromatography using a CarboPac MA1 column (4 × 250 mm; Dionex, Sunnyvale, CA, USA) operated at 30°C and isocratically eluted with 0.6 N NaOH at a flow rate of 0.4 ml min^−1^. WSCs on both systems were quantified *in silico*, using the Chromeleon v. 6.4 software package, against a series of 5 nmol of standard sugars. The quantity of standard sugars used corresponds to the linear response range of the both chromatographic systems.

## Results

Fractions of WSC extracts from desiccated (5% RWC) *C. plantagineum* leaves were collected after separation on an HPLC column. These fractions represented WSCs which eluted like the commercially available RFO standards Raf, Sta, and Ver. They were hydrolyzed with an *Aspergillus niger* acidic α-galactosidase specific for the cleavage of the α1,6-galactosidic linkages in RFOs. Both the standard RFO preparation (Raf, Sta, Ver) and the collected fractions were hydrolyzed to Suc and Gal (Figure 3).

**Figure 1:**
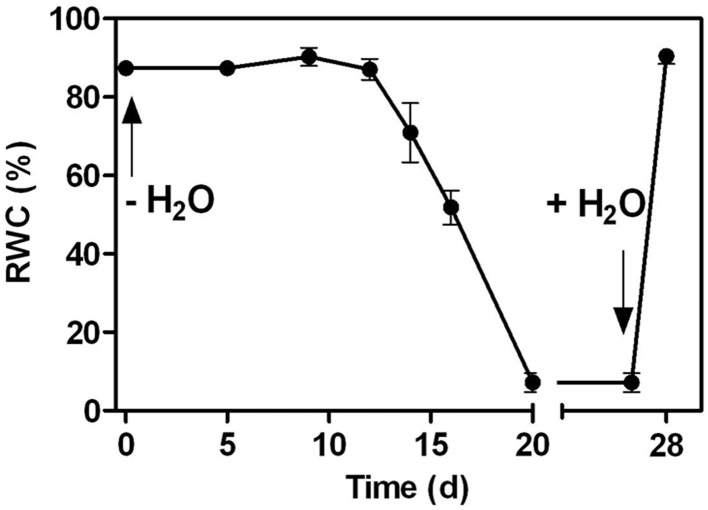
**Changes in the relative water content (RWC) in the leaves of ***C. plantagineum*** plants subjected to a 20 d water deficit stress**. Plants were then held in a dried state for 7 d and water deficit was alleviated by re-watering the plants. Error bars indicate the standard error between the mean of six replicates.

**Figure 2:**
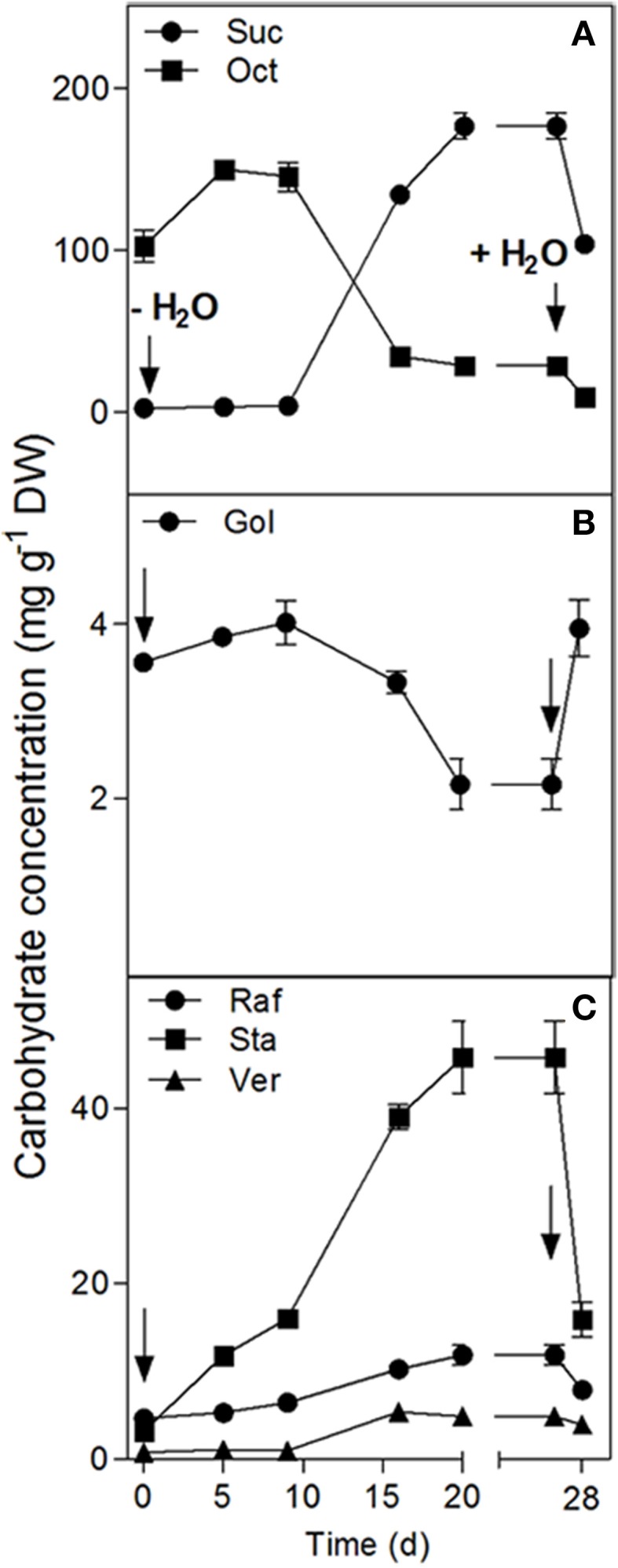
**Changes in the water soluble carbohydrate concentrations in the leaves of ***C***. ***plantagineum*** plants subjected to a 20 d water deficit stress**. Plants were then held in a dried state for 7 d and water deficit was alleviated by re-watering the plants. WCSs were analyzed by HPLC-PAD using MA1 and BC100 columns. Error bars indicate the standard error between the mean of six replicates. Suc, sucrose; Oct, octulose; Gol, galactinol; Raf, raffinose; Sta, stachyose; Ver, verbascose.

A water deficit was imposed on *C. plantagineum* plants over 20 d during which leaf RWC decreased from 87.3% (full turgor) to 7.2% (desiccated state, Figure 1). After a 7 d period in the desiccated state, plants were rewatered and regained their original hydration state reaching a leaf RWC of 90.0% within 24 h (Figure 1). During water deficit *C. plantagineum* leaves showed the classical linear increase in Suc reaching a concentration in leaves of 176.7 mg g^−1^ DW in the desiccated state (Figure 2A). Similarly, the classical inverse relationship of 2-Oct concentration to Suc was observed (Figure 2A). Subsequent (24 h) to rewatering, the leaf Suc concentrations rapidly decreased to 103.8 mg g^−1^ DW, but the Oct levels remained low (9.3 mg g^−1^ DW; Figure 2A). We observed linear increases in the concentrations of the RFOs Raf, Sta, and Ver during water deficit with Sta showing the largest mass increase (from 3.2 to 45.7 mg g^−1^ DW at 5% RWC, Figure 2C). Concentrations of Sta in the desiccated leaves were 3.8-fold higher than Raf and 9.3-fold higher than Ver (Figure 2C). RFO accumulation correlated with a decrease in Gol (3.6–2.2 mg g^−1^ DW at 5% RWC; Figure 2B). As with the concentrations of Suc, those of the RFO members decreased within 24 h after rehydration.

GolS, RafS, and StaS activities decreased strongly (from 1087 to 372 nkat g^−1^ DW for GolS, from 133.6 to 10.1 nkat g^−1^ DW for RafS and from 110.2 to 18.2 nkat g^−1^ DW for StaS), but increased again following 24 h rehydration (Figure 4). Interestingly, the GolS activity decrease correlated with a decline in leaf Gol concentrations (Figure 2B) while RafS and StaS were inversely correlated with the accumulation of Raf and Sta (Figure 2C).

**Figure 3:**
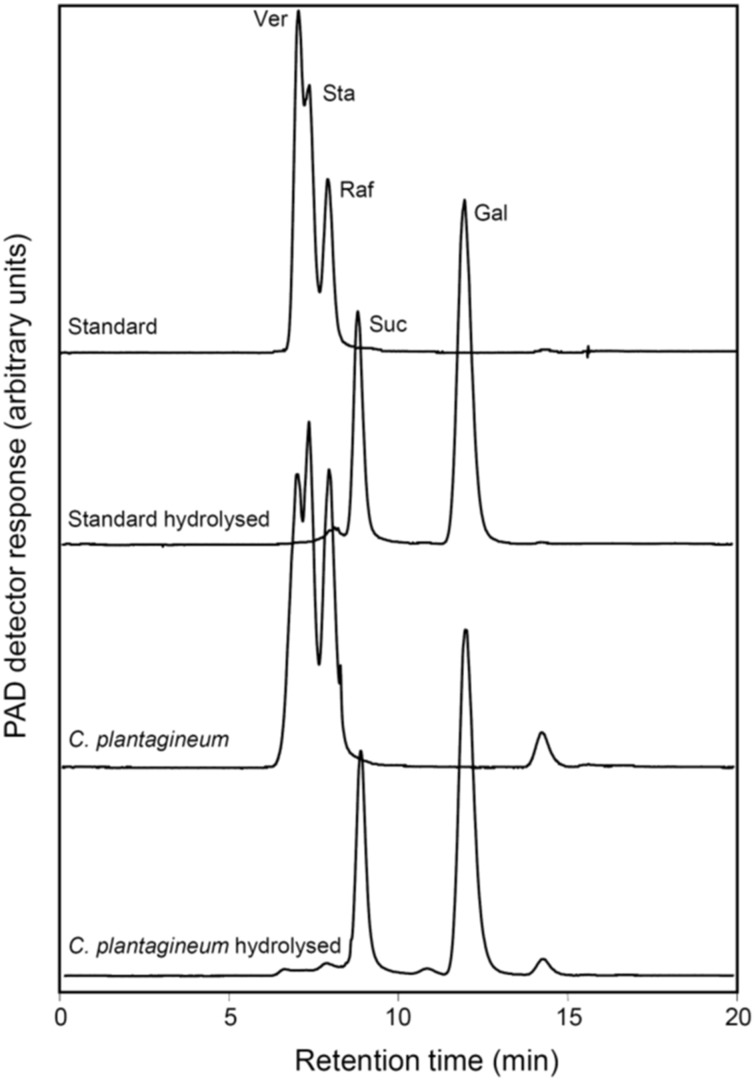
**HPLC-PAD chromatograms representing the identification of the oligosaccharides formed in leaves of water deficit-stressed ***C. plantagineum*** plants (collected at 5% RWC)**. The oligosaccharides were collected, enzymatically hydrolyzed with a commercial alkaline α-galactosidase from *A. niger* and compared with a Ver, Sta, and Raf standard (2 mg ml^−1^ each) treated the same way. Ver, verbascose; Sta, stachyose; Raf, raffinose; Suc, sucrose; Gal, galactose.

**Figure 4:**
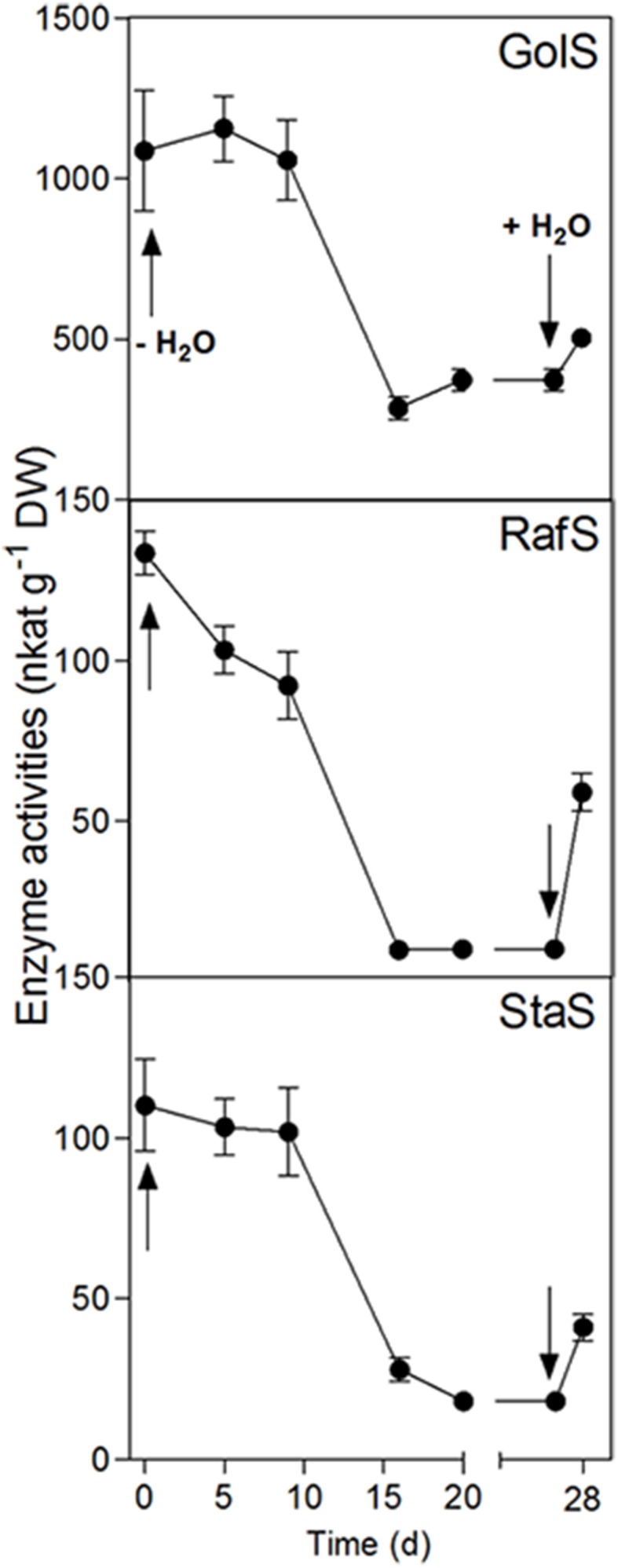
**Enzyme activity changes in the leaves of ***C. plantagineum*** plants subjected to a 20 d water deficit stress**. Plants were then held in a dried state for 7 d and water deficit was alleviated by re-watering the plants. Enzyme activities were analyzed by HPLC-PAD using a BC100 column. Error bars indicate the standard error between the mean of six replicates. GolS, galactinol synthase; RafS, raffinose synthase; StaS, stachyose synthase.

The comparison of the leaf WSC profiles at full turgor or in the desiccated state between three *Craterostigma* species (*C*. *plantagineum, C. agnewi, and C. pumilum*) showed that RFO concentrations (Raf, Sta, and Ver) were increased in the desiccated leaves of the three species. However, desiccated leaves of *C. plantagineum* accumulated about two-fold more Raf and Sta compared to desiccated leaves of *C. agnewi and C. pumilum*.

## Discussion

The resurrection plant *Craterostigma plantagineum* is arguably considered as the research model to elucidate the molecular mechanisms which underpin the unusual ability of resurrection plants being able to survive complete anhydrobiosis. A number of pioneering studies have revealed the multicomponent strategies followed by these plants to ameliorate the effects of water loss (for reviews see Gechev et al., 2012; Dinakar and Bartels, 2013). These include leaf folding mechanisms, induction of antioxidant pathways, de-regulation of seed specific protectants into desiccated leaf tissues and accumulation of secondary metabolites and WSCs. An unusual feature of *C. plantagineum* is the well-characterized accumulation of Suc in leaves undergoing water deficit (Bernacchia et al., 1996). It occurs via an interconversion between the C8 ketose-sugar 2-octulose (present in hydrated leaves). The novelty of such a pathway may have led to the possible contributions of other WSCs in desiccated leaves being largely overlooked.

The leaves of *C. plantagineum* have been shown to have an active RFO biosynthetic pathway up to Sta, at full turgor (Norwood et al., 2000). In that study, Sta was shown to be predominant phloem-mobile sugar accumulating in roots where it was the most abundant storage carbohydrate (>50% of total root WSCs). Under conditions of water deficit the only study that has reported on RFOs investigated *C. plantagineum* roots (Norwood et al., 2003). In that study, it was suggested that under water deficit the Sta store is (i) mobilized in the roots and accumulated as Suc and (ii) is transported from the roots to other areas of the plant to fuel carbohydrate metabolism during tissue desiccation. The re-mobilization of root Sta stores argues for the action of an α-galactosidase that would hydrolyze Sta to Suc and galactose (Gal).

In our study, desiccated leaves (5% RWC) of *C. plantagineum* contained a series of WSCs (not occurring to the same extent in control leaves) that were confirmed to be the RFOs Raf, Sta and Ver (Figure 3). However, enzyme activities for the major RFO biosynthetic enzymes (GolS, RafS, StaS, and VerS) showed a negative correlation to the accumulation of the RFOs over a water deficit period of 28 d (full turgor to 5% leaf RWC). Accumulation of WSCs in resurrection plant leaf tissue undergoing water deficit seems to occur quite early with RFO accumulation reported to occur at leaf RWCs of just below 70% in *Xerophyta viscosa* (Peters et al., 2007) and *Barbacenia purpurea* (Suguiyama et al., 2014), suggestive of a function in osmotic adjustment. Negative correlation of GolS activity to Gol and RFO accumulation was also reported for *X. viscosa* (Peters et al., 2007). We suggest that enzyme activity measured in leaf tissue during the desiccation process is actually sufficient for the accumulation of RFOs we observed over the same period. While the accumulation of the Suc necessary for RFO accumulation is clear (interconversion from 2-octulose and possibly mobilization from the root Sta stores), the source of Gol must be *de novo* (despite a decrease in GolS activity) given that it appears not to be replenished (Figure 2A) but utilized to synthesize RFOs (primarily Sta) during desiccation.

**Figure 5:**
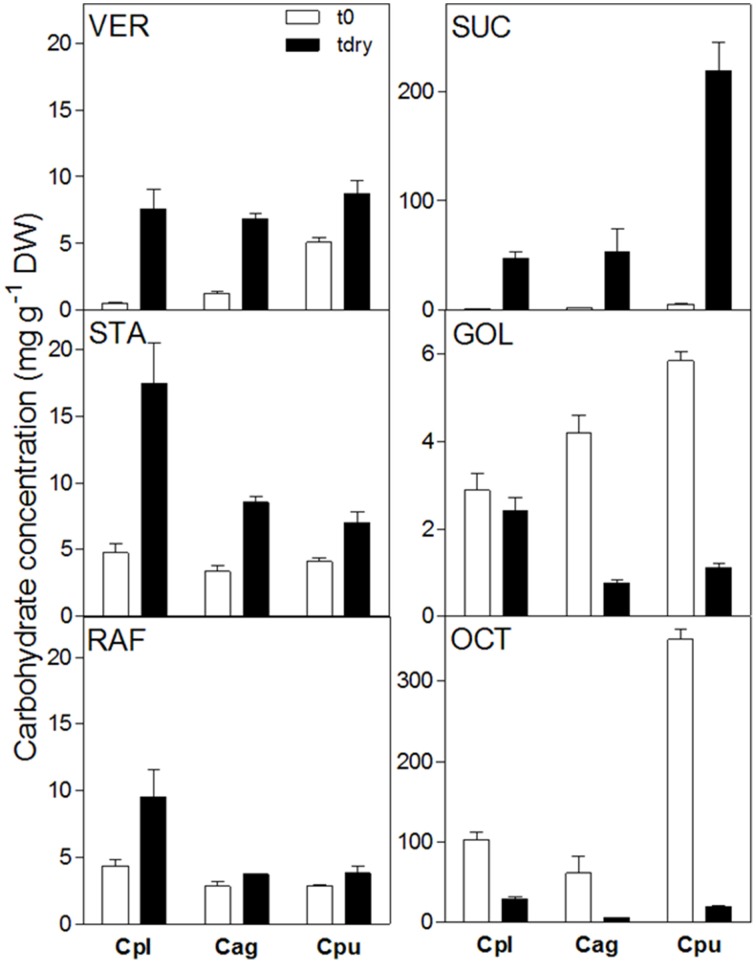
**Comparison of water soluble carbohydrate concentrations in the leaves of ***C***. ***plantagineum***, ***C. agnewi***, and ***C. pumilum*** plants in the fully hydrated state (t0) and the 5% RWC dried state (tdry)**. WSCs were analyzed by HPLC-PAD using MA1 and BC100 columns. Error bars indicate the standard error between the mean of six replicates. Suc, sucrose; Oct, 2-octulose; Gol, galactinol; Raf, raffinose; Sta, stachyose; Ver, verbascose, Cpl, *C. plantagineum*; Cag, *C. agnewi*; Cpu, *C. pumilum*.

In a previous study on RFOs in *X. viscosa* (Peters et al., 2007), a GolS isoform was identified from differential cDNA libraries and heterologously expressed in *E. coli* to demonstrate it was enzymatically a *bona fide* GolS. This provided the foundation to further investigate water deficit-induced RFO accumulation in *X. viscosa*. To date, no RFO biosynthetic genes (GolS, RafS, StaS or VerS) have been reported from cDNA libraries of *C. plantagineum* leaves. We thus chose to expand our analyses to include two additional *Craterostigma* species (*C. agnewi* and *C. pumilum*) to ascertain if our observations in the leaves of *C. plantagineum* were consistent in these species, pointing to a conserved mechanism in response to water-deficit (Figure 5). We indeed found that Raf, Sta, and Ver accumulated in desiccated leaves of all three. Unlike observations in *X. viscosa* where Raf was the predominant RFO, Sta appeared as the dominant RFO in desiccated *Craterostigma* leaves accounting for about 50% of total RFOs in the three species. These findings would appear consistent with the previous reports in *C. plantagineum*, where Sta appears to be the preferred RFO and is used in phloem transport and carbon storage in the roots (Norwood et al., 2000). Interestingly, Raf which is the most common RFO proposed to exert protective functions, accumulated the least in desiccated leaves accounting for about 27.6, 19.6, and 19.5% of the total RFOs in *C. plantagineum, agnewi*, and *pumilum*, respectively.

Despite multiple reports of correlative RFO increases in response to various abiotic stresses, the mechanism by which they exert their protective functions *in vivo* is unclear. A clear function has arguably only been reported *via* reverse genetic studies in Arabidopsis using a RafS mutant (*RS5*, At5g40390, Knaupp et al., 2011). It was demonstrated that Raf accumulating to the chloroplast during cold acclimation (4°C) specifically acts to protect the photosystems of the thylakoid membranes from damage during freeze-thaw cycles. An intriguing hypothesis arises when one considers the role of Raf accumulation in resurrection plants during leaf desiccation. The disaccharide Suc is well-reported to increase in plant leaves exposed to water deficit and has been suggested to function as a typical compatible osmolyte/solute counter-acting the negative effects of cellular water loss. However, if resurrection plant leaves exist in a state of complete anhydrobiosis following desiccation, this implies that Suc will be in a crystalline state. Apart from *in vitro* studies which demonstrated the efficacy of RFOs (Raf, Sta, and Ver) in protecting artificial liposomes from desiccation induced-damage (Buitink et al., 2000; Hincha et al., 2003, 2008), a single report has outlined the ability of small quantities of Raf preventing the formation of the Suc crystal (Caffrey et al., 1988). We believe that small mass increases in RFOs (relative to Suc) may actually be important in the context of resurrection plants, preventing/reducing physical damage that crystalline Suc may cause (for e.g., to cell membranes), when it accumulates to high concentrations in desiccated leaves.

In summary, we have demonstrated that, apart from the very well-reported desiccation-induced inter-conversion of 2-Oct to Suc in *C. plantagineum* leaves, *de novo* synthesis of RFOs occurs. These RFOs accumulate up to the pentasaccharide Ver (Suc-Gal_3_). The most predominant RFO in desiccated leaves is the tetrasaccharide Sta (Suc-Gal_2_). Our findings suggest that while Sta may be remobilized from the roots during desiccation, its appearance in leaves undergoing desiccation is due to *de novo* synthesis (along with other RFOs) to exert an additional carbohydrate-based protection in desiccated leaves.

## Conflict of interest statement

The authors declare that the research was conducted in the absence of any commercial or financial relationships that could be construed as a potential conflict of interest.
